# Valuing and Sustaining (or Not) the Ability of Volunteer Community Health Workers to Deliver Integrated Community Case Management in Northern Ghana: A Qualitative Study

**DOI:** 10.1371/journal.pone.0126322

**Published:** 2015-06-16

**Authors:** Karen Daniels, David Sanders, Emmanuelle Daviaud, Tanya Doherty

**Affiliations:** 1 Health Systems Research Unit, South African Medical Research Council, Cape Town, South Africa; 2 School of Public Health, University of the Western Cape, Bellville, South Africa; 3 School of Child and Adolescent Health, Faculty of Health Sciences, University of Cape Town, Rondebosch, South Africa; University of Oxford, KENYA

## Abstract

**Background:**

Within the integrated community case management of childhood illnesses (iCCM) programme, the traditional health promotion and prevention role of community health workers (CHWs) has been expanded to treatment. Understanding both the impact and the implementation experience of this expanded role are important. In evaluating UNICEF’s implementation of iCCM, this qualitative case study explores the implementation experience in Ghana.

**Methods and Findings:**

Data were collected through a rapid appraisal using focus groups and individual interviews during a field visit in May 2013 to Accra and the Northern Region of Ghana. We sought to understand the experience of iCCM from the perspective of locally based UNICEF staff, their partners, researchers, Ghana health services management staff, CHWs and their supervisors, nurses in health facilities and mothers receiving the service. Our analysis of the findings showed that there is an appreciation both by mothers and by facility level staff for the contribution of CHWs. Appreciation was expressed for the localisation of the treatment of childhood illness, thus saving mothers from the effort and expense of having to seek treatment outside of the village. Despite an overall expression of value for the expanded role of CHWs, we also found that there were problems in supporting and sustaining their efforts. The data showed concern around CHWs being unpaid, poorly supervised, regularly out of stock, lacking in essential equipment and remaining outside the formal health system.

**Conclusions:**

Expanding the roles of CHWs is important and can be valuable, but contextual and health system factors threaten the sustainability of iCCM in Ghana. In this and other implementation sites, policymakers and key donors need to take into account historical lessons from the CHW literature, while exploring innovative and sustainable mechanisms to secure the programme as part of a government owned and government led strategy.

## Introduction

Community health workers (CHWs), whether paid or unpaid have become a part of primary health care (PHC) service delivery in many countries, particularly over the last four decades. This cadre of health workers are well recognised for their ability to make an important contribution to the delivery of key interventions within health systems worldwide [1, 2]. The nature of this contribution has however shifted over the course of time and has been influenced by alternative conceptions of PHC [3, 4]. It is argued that earlier, more comprehensive approaches to PHC recognised that health is determined by broader political and economic forces and thus CHW programmes emerging out of this ethos had a strong community mobilisation mandate [3, 4]. In contrast later, more selective approaches to PHC, have been more disease focused, and within this approach CHWs have become predominantly health extension workers rather than community advocates [3, 4].

Historically, CHWs have been engaged more frequently in health promotion and disease prevention tasks than in curative and treatment functions [5]. These latter functions have been seen as the purview of formally trained health workers. However, health systems are coming under increased strain [6, 7]. This includes difficulties in managing growing epidemics of infectious diseases, notably HIV/AIDS, difficulty in attracting and retaining formal health workers especially in rural and under resourced areas, and an ever pressing need by governments and multilateral agencies to meet the Millennium Development Goals (MDGs) [8]. In the face of these challenges the roles of CHWs are increasingly being reconceptualised to allow them to take on additional functions in the public health sector. Thus there has been much discussion about the concept of task shifting from one cadre of health workers to another, and indeed for creating new cadres of health workers [9, 10]. Within this discussion it is widely agreed that CHWs can successfully, safely and effectively take on a range of curative and treatment functions [1]. One international treatment approach that has adopted an expanded role for CHWs is integrated community case management of childhood illnesses (iCCM) [11–14]. Within this approach CHWs operating at a community level (e.g. by visiting homes in a rural village or establishing a health post), deliver treatment for pneumonia, diarrhoea and malaria. This treatment includes the administration of antibiotics, oral rehydration solution and zinc, rapid diagnostic tests for malaria and artemisinin combination therapy. In Ghana, where our study was based, iCCM has been implemented as part of a broader UNICEF programme of Integrated Health Systems Strengthening (IHSS) support through the Catalytic Initiative to Save a Million Lives [15, 16]. The main focus of the IHSS support was to strengthen the health system to prevent and provide effective treatment, especially at community level, for the main causes of child deaths.

Despite Ghana being reclassified from a low income country to a lower middle income country, the economic prosperity experienced after the discovery of offshore oil has not trickled into the public health system which is still heavily dependent on donor support [17–19]. In 2003, in an attempt to replace a system of user fees for health care, Ghana introduced a National Health Insurance Scheme (NHIS) through a legislative Act of Parliament (The NHIS Act) [20]. Scale up of the system began in 2004 and it reached full operation nationally in 2006. The NHIS is largely funded through a NHI levy, which is an additional 2.5% value added tax (VAT), while the remainder is gathered through pay-roll deduction and premium and registration fee payment. All children under 18 years are exempt from premium payments and NHIS registration is free for children under 5, pregnant women and people over 65 [21]. For working people in formal employment the fee is deducted at source.

The NHIS covers inpatient and outpatient services, essential drugs, maternity care and emergency care. Preventive services such as immunisations or family planning are not included in the NHIS although the Ghana Health Services (GHS) is a GAVI recipient. At present, most of the Ghanaian health budget is directed towards staff costs, with medicines and supplies mostly being covered by donors [17]. But despite this initiative, paid health care staff still cannot reach all communities, especially rural communities. Currently rural health care is delivered through the Community-based Health Planning and Services (CHPS) which originated in the late 1970s, but gained momentum in the 1990s [22, 23]. This service is staffed by mid-level workers known as Community Health Nurses (CHN) or Community Health Officers (CHO). For this system to work ideally it is dependent on the construction in close proximity to rural villages, of community health compounds, from which the CHO or CHN operate and in which they can live. But this ideal has not been realised due to difficulty in the construction of the compounds and in the recruitment of rural staff. Thus many CHPS zones do not have compounds with functioning facilities or resident CHO staff, resulting in community members having to travel to health centres or even hospitals for care when outreach services are not present. To complement the existing workforce, the delivery of health care in rural Ghana includes making use of the services of CHWs known as community based agents (CBAs). Such workers have been a part of the Ghanaian health system since the 1970s [22]. While there is a long history of task shifting in Ghana, it seems to have been focused on the creation of intermediate professional staff levels [24–26]. Despite similar challenges as experienced elsewhere in the world (such a professional gate keeping) [25, 27] task shifting is now accepted as part of the national human resources for health plan [28]. However, this plan seems to extend only to the CHPS level, without a formal place for CBAs who are regarded as volunteers [27].

In general, CBAs in Ghana are volunteers who do not receive any government salary [27]. The CBAs in the evaluated intervention were wholly community workers since they operate at village level and the process of recruitment and selection engages the whole community [15]. CBAs offer preventive and curative services which they deliver directly to the community (e.g. at clients’ homes) rather than from health posts (CHPS). The curative services which they offer are however not covered by the NHIS and clients therefore have to pay a token amount for medicines received from CBAs. Half of this token is paid back to the CBA and is regarded as an incentive in lieu of a salary. In the three Northern Regions of Ghana, where this study took place, CBAs are approximately 50% female [29]. The intervention policy is to have one male and one female CBA for each community [15, 29]. Most CBAs are illiterate with very little schooling; their basic training to become a CBA is 5 days [29].

In 2009 the child health policy and child health strategy were revised to include treatment with amoxicillin (for pneumonia) and zinc (for diarrhoea) by trained CBAs. This paved the way for implementation of the full iCCM package. In Ghana, the CBAs are the frontline workers in the delivery of iCCM, but until the 2009 change in government policy, they were not allowed to deliver treatment. In this qualitative study we explore the implementation of iCCM in the Northern Region of Ghana. We focus here on how these CBAs with their expanded roles were valued, sustained and supported within the existing health system.

## Methods

This descriptive qualitative study [30] by rapid appraisal [31, 32] of the IHSS intervention in Northern Ghana forms part of a larger evaluation of the intervention in 6 African countries [29]. This broader evaluation encompasses both quantitative measures of coverage, impact, effectiveness and costs as well as qualitative exploration of implementation experience in each country. The full set of reports, including methods for the broader evaluation, can be found at http://www.mrc.ac.za/healthsystems/publications.htm.

### Study intervention

Between January 2008 and 31 May 2013 UNICEF, with funding from the Department of Foreign Affairs, Trade and Development, Canada (DFATD), supported the implementation of the IHSS programme in four regions of Ghana (Central, Upper East, Upper West and Northern) [15]. The aim of the programme was to support the High Impact Rapid Delivery (HIRD) strategy of the Government of Ghana, which began in 2007 and aimed to increase access to evidence-based high impact interventions to reduce maternal and child mortality [15]. During phase one of the IHSS the focus was on providing support for strengthening immunisations, vitamin A supplementation, infant and young child feeding, procuring and distributing insecticide treated nets, training and quality improvement. Implementation of iCCM of diarrhoea, malaria and pneumonia started in late 2010.

### Data collection

Qualitative data were gathered through rapid appraisal [31, 32] during a 9 day country visit to Ghana which took place in May 2013. The data were collected by three senior researchers (KD, TD and ED), all of whom are women. Collectively they have training in social science research methods, public health and health systems research. The researchers engaged in individual interviews, focus group discussions and field visits [30, 33–35]. This involved speaking with key informants in Accra and Tamale (capital of the Northern region), as well as visits to local health centres and villages surrounding Tamale.

Where necessary (in interviews with mothers, CBAs and CBA supervisors), the services of interpreters were used. Although the interpreters were provided by the GHS, several of the interviewees understood English well enough to check the accuracy of the translation. All interviews took place either at the offices of the interviewees, at a district office or health centre, or in the communities. Interviews were audio recorded and the researchers took field notes. None of the interviews or focus groups were repeated.

### Participants and sampling

In advance of the country visit we sent a proposed list of interviewees to the UNICEF country team, who then assisted with pre-scheduling appointments. In compiling this list we gave consideration to gaining as wide a range of opinion as possible so as to ensure a fair representation of how the implementation of iCCM was experienced in Ghana [36]. In choosing the health centres and villages we ensured representation between sites that were close to Tamale town centre as well visiting remote villages where access to all services was poor. Informants included UNICEF staff (9) and other partners and researchers (5), national and regional GHS staff (13), CBA supervisors/zonal coordinators (6), nurses in health facilities (11), CBAs (24) and mothers (37) and one village chief (1) [Table 1]. The zonal coordinators were all older men (40 years and older) who had been previously engaged as CBAs in guinea worm eradication. None of those we interviewed had jobs beyond the intervention and they did not generate their own income outside of the intervention. The CBAs were specifically engaged for this intervention, with all of them having 4 years experience. All of those we interviewed generated their own income outside of the intervention including being farmers, petty traders, and carpenters. They were younger than the zonal coordinators, with ages ranging from 21 years old to 32 years old. Of those we interviewed 15 were male and 9 were female. The mothers who participated had an age range of between 20 and 40 years old, with between 1 and 11 children.

**Table 1:** Summary of participants.

	Participant category	Number of interviews
**Individual interviews**	Ghana Health Services (GHS)	8 male, 5 female
	Partners and researchers (P/R)	1 male, 4 female
	Nursing staff – includes midwives, community health nurses, community health nurses, enrolled nurses, registered nurses (N)	2 male, 9 female
	Village chief	1 male
	UNICEF Country office	2 male, 3 female
	UNICEF Regional office (UNICEF)	2 male, 2 female
**Focus Groups**	CBA supervisors – zonal coordinators (ZC)	6 male
	CBAs (2–8 per group)	15 male, 9 female
	Mothers (8–13 per group) (M)	37 female

### Data analysis

On the last day in the field an initial reflection of our insights was presented to UNICEF staff. Thereafter we conducted a simple manifest analysis of the qualitative material [30, 37]. Since this was not an ethnographic study we were simply interested in what happened and what was experienced rather than trying to understand the deeper meaning of the experience. Exploring such meaning was not our evaluation intention and would have required a different study design. We analysed the data both deductively and inductively [38]. Deductively, we sought to find answers to predefined questions (e.g. how did this intervention fit within the policy environment? or, what evidence was there of health systems strengthening on the ground?). Inductively, we tried to understand what new information and insights could be gleaned from the interviews and our experiences of visiting the field.

The analysis was based on the typed interview and focus group notes as well as reflections from the field. This material was repeatedly reviewed by KD and TD. We annotated our reflections while reading, and then came together to discuss, compare and critique our insights. Based on this analysis the data were electronically (using a word processor) grouped into categories, the results of which are reported in narrative form in this paper.

### Ethics

This study received ethical approval from the South African Medical Research Council (EC026-9/2012). The interview and focus group processes, including the consent procedures, were also approved by the ethics committee.

Before engaging with participants we explained in detail who we were, why we were visiting and why we wanted to speak with them. When necessary, especially with community members, CBAs and their supervisors, we used the services of a translator to explain our research aim and the consenting process. In all cases we tried to ensure that participants understood what we were asking them to agree to, and what their rights were, especially the right not to participate. Where participants were literate we obtained signed informed consent from them. For those who were not, consent was obtained orally. Since we could not record the oral submissions, we allowed participants the opportunity to leave before we started the audio recording. However, as has been our previous experience, no one left beforehand but occasionally participants would leave during the interview. We were guided by UNICEF and GHS field staff as to when it was necessary to obtain permission from community leaders such as the chief, and in such instances included their opinions on iCCM as part of our data.

## Results

### CBAs as a perceived valuable resource

Our data showed that CBAs were valued on the ground for the contribution that they brought to the local health system. Their work in supporting outreach clinics was appreciated both by UNICEF informants and by facility staff. According to UNICEF,

*“CBAs assist with mobilising people for child welfare outreach clinics*, *National Immunisation Days as well as community-based management of acute malnutrition”* (UNICEF).

Interviews with facility-based staff revealed that they valued the work of the CBAs, especially in supporting facility outreach activities,

*“The CBAs are a real help*. *They mobilize mothers for activities*. *They identify critical cases and refer*. *They are there at every outreach”* (N).

Another nurse shared how CBAs are helping their communities but described how it took time for communities to trust their skills,

*“they provide a good quality service*. *They are saving mothers from having to travel*. *Mothers didn’t know enough about CBAs and didn’t trust their skills*. *They did the last refresher training in the community so people could see them and see their training*. *They are people saving their own people”* (N).

There was also some concern from facility staff that CBAs were not sufficiently literate,

“*some of them are good*. *They are doing well*, *but illiteracy is a problem*. *Education would help them a lot”* (N).

In contrast to the occasional hesitation by facility-staff the mothers we spoke to uniformly appreciated the presence of CBAs in their communities,

*“CBAs are very affordable*, *instead of carrying a child to Saboba and spending more money there—we have to eat*, *have to buy food for the child”* (M).

Another mother explained that, *“If a child is sick we take him or her to the CBAs house”* (M). Mothers also appreciated the proximity of the CBAs

*“The CBAs live in the village so we see them every day*. *They make a follow up visit the day after they have given treatment”* (M).

### CBAs as advocates for infrastructural resources

In the context of poor infrastructure, particularly sanitation, CBAs contributed to health promotion at a community level,

*“if you look at the three northern regions*, *you will see that they have the worst sanitation rates yet cholera is limited due to the work of the CBAs”* (UNICEF).

In the face of this poor infrastructure, we were also told that they acted as advocates in assisting their communities to push for and acquire the infrastructural resources needed to live healthily. But two contrary perspectives were offered on their success in this regard. One mother explained,

*“The CBAs have been lobbying around clean water*. *Now we are taking water from the dam*. *Also having better toilets*. *This community uses the public toilets at the school”* (M).

But, the perception that CBAs were able to successfully advocate for better infrastructure was not uniformly shared. A different mother described the huge needs in her community for improvement in basic infrastructure,

*“we need mosquito nets*, *drinking water- there is no stand pump here*. *We get water from rivers*. *We boil it and then put it in water pots*. *We are waiting for an NGO to bring us a stand pump*. *The CBA can’t help with that*. *We practice open defecation*. *There is food insecurity at certain times of the year*.*”*(M).

Thus, although there may have been a desire for CBAs to act as advocates, from what we heard, their ability to enable change was not uniform and support from higher levels, such as from NGOs, was still seen to be required.

### CBAs or CHNs

The deep sense of appreciation for CBAs which we heard from people on the ground was not as widely expressed at higher levels. Instead, at the national level some scepticism was expressed regarding the sustainability of the CBA programme as well as its relevance. Some informants felt that greater focus should be spent on training more CHNs instead of CBAs. As one informant from GHS explained

*“If we had more CHNs and they worked well*, *then we would need less CBAs”* (GHS).

A Ghanaian researcher of longstanding shared this sentiment and went on to say that CBAs must be formally linked to the health services,

*“We’re glad we had an interim measure*, *the community also saw it as an interim measure*. *Now our idea was that these village health workers are progressively upgraded*, *because what we want are trained nurses*, *we want trained nurses*, *we understand what you are saying about primary health care*, *but our needs don’t end there*. *If we have an emergency in this community*, *our problem has always been that despite your village health worker*, *I will probably die*. *There should be some link between the village health worker and higher levels of services”* (R).

### CBAs as an unpaid resource

We also found that the CBAs we met expressed altruistic appreciation for being allowed to be a part of the programme, and be able to assist their communities.

*“We have come to help them*. *When I have stock I will go around and tell people*. *Sometimes people come and call me*. *Outreach from the CHPS comes monthly*. *The day before the nurse sends a message to me and I go around telling people to wait for the outreach”* (CBA).

Yet, some informants felt that their service was not valued in a tangible way, such as through a financial incentive, or through a place within the formal government health system,

*“CBAs are complaining about needing to be paid*. *There is no refund to the CBAs when they use their own phones*. *When I have something in my bag I give them”* (GHS).

“*people don’t value things that are free*. *CBAs want recognition most of all*. *Everyone needs to understand that we must recognize and motivate them”* (GHS).

*“Volunteers are everywhere but they are not being recognized*. *They have no official place in the GHS”* (UNICEF).

There were further concerns that the lack of incentives caused CBAs to withdraw from the programme to meet their own livelihood needs.

*“Sometimes CBAs become dormant if they don’t get any incentives*. *During the farming season they go to their fields and they are not available*. *They may go and live on their farms for several weeks”* (UNICEF).

As it was, CBAs split their time between programme activities and other income generating activities such as farming and petty trading. One CBA described a typical day,

*“Between 4*:*30–6*:*00AM I clean the house*, *take care of household chores*, *from 6*:*00–12*:*30 I do home visits and treatment*, *from 12*:*30PM onwards I conduct my own trading”* (CBA).

Several other CBAs told us of a similar split between dedicated community time and dedicated time for income generation. Based on our examination of CBA registers which showed poor utilisation of their services, it appeared that the services they offered were far more ad hoc than our respondents suggested, with CBAs responding to calls for assistance, rather than going out and looking for cases. Furthermore, in visiting homesteads and driving through villages, it was clear that rural poverty is widespread, and that it would be difficult for anyone to volunteer at the expense of generating some kind of income because it seems unlikely to us that the collective homestead would be able to sustain a full-time volunteer.

### Sustaining and supporting: Remaining on the outside

#### Poorly supported general supervisors

CBAs receive two forms of supervision, general and clinical. The general supervision is conducted by a group of men known as zonal coordinators. These men had been chosen for the task of CBA supervision because of their previous experience as CBAs in guinea worm eradication programmes. When guinea worm was eradicated from Ghana in 2010 their role changed to supervising CBAs. They are allocated to health zones and each co-ordinator has between 20 and 40 CBAs to supervise.

Like the CBAs the zonal coordinators are not part of the formal government public health service and are not paid. Unlike the CBAs, these co-ordinators do not have any other jobs and since becoming supervisors have lost their previous remuneration,

*“The zonal co-ordinators used to get allowances (20 cedi a month) when they were doing guinea worm surveillance*. *Now they don’t really get anything regularly”* (UNICEF).

Since the end of the guinea worm programme the zonal co-ordinators receive piece-meal incentives irregularly such as a bar of soap at the end of the month. A district director described how zonal co-ordinators are incentivized in his district,

*“Quarterly meetings are a motivation for zonal coordinators*. *They receive 20 Ghana cedi for lunch*, *in cash*, *which they can use for whatever they like (not necessarily to buy lunch with)*. *When there are other programmes then they are involved in the campaigns and get paid for that”* (GHS).

Our interviews with zonal coordinators showed a combination of a strong sense of altruism from witnessing the positive health benefits for their communities resulting from previous health campaigns, as well as a strong emphasis on their needs. However, the focus on their needs seemed to describe what they needed to do the job more effectively and not as might have been expected, the personal need for remuneration. They described their needs especially in relation to transport and other requirements to do their job,

*“we need motivation – allowances*, *motorbikes*, *uniforms*, *identification badges*, *baskets to carry drugs in (wooden boxes are too heavy for the bicycles)*, *rain coats and boots for the rainy season”* (ZC).

Another zonal co-ordinator confirmed these needs,

*“At the end of each month we gather at the health centre*. *We were given bicycles to use*. *All of them are now broken*. *We now use our own personal bicycles”* (ZC).

A district director also confirmed these needs for which they do not have a budget,

*“We also need boots*, *rain coats*, *torch and bicycles for the zonal co ordinators*. *These have only been given once in 2007 when the programme started”* (GHS).

#### Poor capacity for clinical supervision

Clinical supervision is the responsibility of CHOs/CHNs who are trained in iCCM. Observation of CBA case management is supposed to be done once or twice a year using tools developed by UNICEF. However our interviews in the field raised problems with the level of supervision actually given. During our time in the field we were told that there was a high turnover of CHO/CHNs (perceived to be between 30 and 40 percent annually). This, we were told, created a major problem for consistent and coherent CBA supervision because of the loss of trained supervisors, who were then replaced by untrained new incumbents. Thus, as we understood, many of the health workers doing the supervision had not received iCCM training.

#### Poor supply of stocks

A senior official in GHS shared that, “*The supply (of ACT) has been quite erratic”* when asked why she responded that procurement was a problem. Drug stock outs were reported to be a major problem by all levels of informants. A health facility a nurse informed us, *“ACTs are currently out of stock for one and a half months*. *80% of districts are out of stock of ACTs currently”* (N). A CBA confirmed the same problem,

“*I hardly ever run out of stock but today I have only 3 doses of ACTs left*. *2 weeks ago I got new stock of ACTs but now I have only 3 doses left*. *I have no ORS sachets*. *I ran out a week ago*. *I get supplies from the health centre*. *A man in the community calls the health centre*. *They will send the supplies with someone to me*. *I don’t pay the man to use his phone*” (CBA).

A mother described what happens when the CBA has no drug stocks, *“If the CBA runs out of drugs we go to the health centre*, *it is 2 hours away and we walk”* (M). Another mother shared the same concerns, *“The biggest problem is stock outs- mostly for malaria tablets”* (M). Drug stock outs are so common that the CBAs inform their community when they have stocks as one mother stated,

*“The CBAs tell us when they have medicine in stock*. *Before the CBAs were working we used to go straight to the hospital”* (M).

A village chief explained how CBAs need transport to assist them with collecting drugs from the health facility,

*“When the CBA runs out of stock they have to travel far to get stock on foot*. *CBAs were given bicycles*, *not good quality*, *now they are broken down*. *They need cars or motorbikes”* (Village chief).

From our discussions and our experiences in the field we learnt that certain drugs are available at the community level from licensed chemical sellers. These sellers are recognized by the pharmacy council. We understood that they were selling ACTs, amoxicillin and cough syrups amongst other things. They are mostly men and their supervision was perceived by our respondents to be sporadic. The licensed chemical sellers are entirely private and for profit and not linked to the NHIS. They mainly operate in hard-to-reach areas where there are no CHPS. From the perspective of some of our respondents there is little control over the quality of the drugs they sell. An informant in UNICEF described the role of the licensed chemical sellers,

*“The first point of call for people when they are sick is the licensed chemical suppliers*. *They have marketing skills*. *There are perceptions that their quality is better”* (UNICEF).

A mother described how she goes to the more expensive licensed chemical sellers when she can’t access the CBA,

*“If the CBA is unavailable and the child has diarrhoea we go and buy ORS from the drug seller who comes on his motorbike*. *If there is no ORS we boil water and add sugar and salt*. *We also give rice water*. *1 sachet of ORS costs 20 pesewas*. *The CBA sells zinc tablets plus 3 sachets of ORS for 70 pesewas*. *We earn between 50 pesewas and 1 cedi a day from farming”* (M).

#### Navigating a means of support while outside of the NHIS

As described in the introduction, CBAs are not part of the formal system and their services are not remunerated by the NHIS. However, as pointed to in the UNICEF project reports, advocating for such inclusion was a clear part of their intervention strategy [15, 16]. Four main perspectives emerged from our data collection in relation to how this exclusion impacted on iCCM and the use of CBAs. Some participants felt that exclusion was a deterrent from the intervention and impacted on its sustainability. Another perspective was that it was not a barrier to accessing treatment, and still a further view expressed was that flexibility could be exercised in ensuring that CBAs were reimbursed. A fourth perspective was that the NHIS was a burden.

The perspective that the exclusion impacted as a current deterrent to mothers accessing iCCM at the CBA level, and that this threatened long term sustainability beyond the intervention, was primarily held by UNICEF and other development partners,

*“It is still a struggle to get CBAs to be included in the NHIS*. *There has been lots of advocacy by UNICEF with the regional NHIS schemes*. *In some districts CBAs are included in the NHIS but it is very unofficial*. *CBAs must take the client’s NHIS card to the local CHPS to claim there as if the child was treated at the CHPS*. *It sometimes takes days for CBAs to get the card to the CHPS*. *In places where the CBAs are getting reimbursed by the NHIS they are very enthusiastic*. *In one district the district director gave the CBAs petty cash while they wait for the reimbursement from the NHIS (in Upper East region)”*(P).

Another partner also echoed this concern, *“iCCM sustainability is threatened if it is not included in the NHIS”*(P). UNICEF staff explained that there was a fear that the inclusion of CBAs would be *“too much too handle”* financially, they also described the NHIS as “*sick*” (UNICEF). But from their perspective they felt that it would easier and cheaper to provide treatment at the community level. They felt that the lack of support to this level created resource constraints.

Two health facility staff also raised concerns about the exclusion on a practical level. They suggested that it acted as a deterrent to the use of CBAs. They felt that mothers who were enrolled in the scheme would bypass the CBAs, opting instead to go directly to the health facilities, where their enrolment entitled them to free service, as compared to having to pay a token fee for services or treatment received from the CBAs. One CHO expressed that the lack of inclusion of CBAs in the NHIS was a deterrent to their utilisation,

*“There is low demand for them – and lack of motivation amongst them*. *Mothers with health insurance don’t want to use them because they have to pay”* (N).

Contrary to the concerns raised above, neither mothers nor CBAs raised the NHIS exclusion and the concomitant resulting token payment to the CBA as being a deterrent. They argued that the CBAs would treat the children even when the parents did not have the money, allowing them to pay later when they were able to do so. This willingness to pay for treatment was attributed by one group of CBAs to a perception that this level of care was cheap in comparison to seeking care from other levels.

In the third perspective offered there seemed to have been a middle road between inclusion and exclusion. As suggested by both UNICEF staff and by regional and district level GHS staff, on this middle road there was some flexibility in the translation of NHIS policy with regard to CBAs. This flexibility resulted in some conflicting narratives as to what was happening on the ground. For example, while staff at one health centre suggested that, *“people insured on NHIS don’t pay the CBA*, *the CBA brings their card to the health centre”* (N), and they would then have their token payments reimbursed. Other participants (mothers and CBAs), suggested a different system in which a token payment was made to the CBAs who then paid this over to the health centre after which they were reimbursed in part (50%). An intervention partner explained that districts found their own ways to motivate the CBAs by facilitating their reimbursement through the NHIS,

“*In the Upper East they keep petty cash to pay the CBAs while waiting for reimbursement from the NHIS*” (P).

A fourth perspective of the NHIS registration as a burden emerged from some mothers and CBAs. CBAs in one focus group suggested that it may be more expensive and cumbersome for insured community members to travel to a health centre for free treatment, than for them to get treatment from the CBA for which they would have to pay a token. Thus they questioned the point of community members being registered.

Overall our interviews did not clarify exactly why CBAs were excluded, and nor did it confirm that this exclusion acted as a deterrent to mothers accessing iCCM from CBAs.

#### So who do they belong to?: Unsustained intervention infrastructure support

Having CBAs outside of the formal health system means that this level of health worker is not “owned” by government. While UNICEF was regarded by our informants as the key driver, government was seen as slow to take ownership,

*“There should be some level of ownership by government for sustainability*. *This is one of the key challenges*. *Government is very slow*. *It is difficult to get consensus from government*. *There should be strong leadership*, *ownership”* (P).

This lack of ownership poses a particular problem for infrastructural sustainability. During our field visit we observed the rural infrastructure as being poor, as it is for most of rural Africa. In the places we visited access to sanitation, electricity, tarred roads, transport and equipment were largely missing. CBAs offered their services from their homes or from the homes of their clients, working with nothing but the rudimentary medical kits supplied by UNICEF, and often having to conduct their services on foot because the bicycles they had been supplied with years ago were by now in serious disrepair. This lack of equipment and supplies to perform their tasks were seen as a challenge by CBAs,

*“Mothers in the community appreciate my work*. *It would help if we had a constant supply of medicines*. *The biggest challenge is transport*. *I also need a cell phone*. *My bicycle is broken*. *A motorbike would be a big help”* (CBA).

*“Once the community is aware that you have the treatment then they’ll wake you*. *We’re woken up in the night when it’s raining and we have no raincoat*, *no boots*, *and the bicycles are spoiled (broken)”* (CBA).

One CBA explained *“we work at night with no lamps*. *We use fire light and people come to the house”* (CBA). Another mentioned the desire to have a uniform, *“We want a form of identification (uniform)*. *This will make us unique and people will know what we are doing”* (CBA).

While the initial supply of these consumables was part of the donor funded intervention, it was unclear as to how their re-supply and maintenance would be managed post intervention, given that the CBAs programme is not owned by government.

## Discussion

This qualitative study by rapid appraisal highlights the challenges and opportunities of delivering a community-based intervention, iCCM, within a complex health system. Within this context there were many factors influencing how iCCM was received.

Health care in this context is provided directly and indirectly by a range of stakeholders, which meant mothers in our study had the option of accessing services for their children from government, through the UNICEF-supported CBAs and through private licensed chemical sellers. From what we were told, mothers favoured CBA care (even when they had to pay a token for it) over more distant care at a health centre or more expensive care from a chemical seller. The addition of the treatment component to the role of CBAs made a difference to how clients valued CBAs. This favourable response by mothers cannot however be taken at face value as a survey of this programme showed poor utilisation of CBAs for curative services [39]. This ambiguity may be related to the overall context of resource and service constraints, where our respondents may, when given the option, choose to seek care higher levels of the health system, but be unwilling in a research interview, to criticise CBA services that they would still need when other care becomes inaccessible.

Similarly to the mothers, respondents from the GHS all favoured the intervention when discussing it during our field visit, but in practice CBAs remain outside of the NHIS and task shifting is only formally implemented to the CHPS level. While we only had one respondent suggesting that nurses may be needed more than CBAs, given the policy of not including CBAs, it is likely that this position was more widely held than we were told. We know that there is a long standing history of nationally generated innovations for improving health care access, pointedly through CHPS and the NHIS. We also know that there is a long standing history of donor partnership and dependence, during which the ability to manage donors and ensure government priorities are met, has been variable [40]. So despite an ethos of national innovation, within a context of financial constraint it might be hard to voice either to evaluating researchers or to implementing organisations, that an intervention is not favoured. Thus as with mothers, the response of the GHS to this intervention can be said to be ambiguous.

This ambiguity may explain some of the challenges we found including:

- Conflicting views about CBA skills ability;
- Multiple systems of reward, not formalised, sometimes circumventing policy;
- Poorly supported general supervision;
- A weak system of clinical supervision;
- Inconsistent drug supply, with mothers sometimes having to access more expensive care;
- Variable reports of CBAs’ ability to act as advocates;
- Lack of clarity in how CBAs and zonal coordinators would be supported, particularly in the resupply of their consumable tools and resources, after the UNICEF support ended.

The concern raised in our interviews about the weakness of clinical supervision was confirmed through a quantitative survey of the intervention [41]. The survey showed that supervision, which includes observation of case management, was very low (6%) and that even where CHOs/CHNs were trained in iCCM, less than half reported supervising CBAs during the past month [41].

Previous research has shown challenges to community based service delivery [11, 42, 43]. Research emanating from the Navrongo experiment and from the Accelerated Child Survival and Development programme, both conducted in the Northern region of Ghana between 2001 and 2005, showed that there were problems with implementing volunteer programmes [11, 22, 23]. A previous study of community based surveillance volunteers in the same region, showed similar to our study, that volunteers desired tangible recognition and resupply of their resources and equipment [43]. Our study, although rapid, suggests that some of these challenges continue.

An historical overview of the CHW literature reveals concerns about inadequate support, unfair expectations, a lack of extrinsic incentives, un-sustained resources, poor planning, poor integration and poor supervision [44, 45]. A growing more recent literature on how to sustain CHW programmes [2, 46, 47], repeats the historical lessons of calling for CHWs to be better motivated, supervised, supported, offered context specific remuneration and incentives [48–51]. The current focus in the literature is on sustainability through the use of incentives, good supervision, good training, context appropriate remuneration, and adequate supply of resources. While the literature is increasingly vocal on the incentivising mechanism (what should be done), ownership of the process of sustaining incentives and interventions overall (who should do it), is less well addressed. Our study points to the importance of clarifying ownership and national buy-in.

We found a new programme adding additional components and tasks to the package of services that CHWs are expected to deliver, without changing the underlying structure of the existing delivery platform. In this instance CHWs, who were traditionally outside the system, remained outside the system. In the drive to achieve MDG 4, iCCM is being implemented in an increasing number of countries, where health systems differ significantly as do social, economic and political contexts. Such contextual differences necessarily influence the implementation, and especially the sustainability of interventions [52]. While analysing and addressing health system factors and contextual constraints may be time-consuming, ensuring such considerations are taken into account in programme design is likely to ensure a more sustained improvement in health care [20]. Previous research has pointed to policy maker support for strengthening of the CHPS programme [22, 23] rather than for lower level CBAs. It may therefore have been important to establish in advance what the national health systems priorities were, how this programme fitted within those priorities and whether policy makers were able to commit to supporting the programme over the long run in terms of ensuring continuing provision of supplies and funding for community-based care. Such discussions may possibly have pointed to different priorities—such as paying more attention to strengthening the capacity of CHOs and CHNs to deliver treatment and by focusing on how workers at that level might be retained over the long term. However, such deliberations may be deflected in a situation where political pressure is accompanied by funding and resources. Previous research in Ghana has shown that aligning national government priorities with donor expectations is no easy task [17]. But such alignment is necessary in order to avoid short term unsustainable projects that are not owned by national government [21]. Our data demonstrate that CBAs and their supervisors are already experiencing a shortage of necessary resources such as drugs and supplies, bicycles, rain gear and torches, all of which require regular replacement. These resource constraints exist even during the period of donor support; it is likely that such constraints will worsen after such support ends [21].

### Strengths and limitations

All of the researchers who visited Ghana are experienced in health systems research. The key strength of this evaluation was that this group of researchers are not in the direct employ of UNICEF and therefore are able to objectively assess the impact, outcomes and experiences of the implementation of IHSS and to see and experience for themselves how the IHSS was implemented. While in Ghana the team spoke to a wide range of stakeholders and were therefore able to gain a composite picture on which to base the evaluation. The field visits also helped us gain some insight into the cultural and political context in which the intervention took place, something that we could not have achieved by merely doing a desk based evaluation. Notwithstanding the above, it is possible that assistance with the selection of informants by the commissioning agency (UNICEF) and their visible involvement in supporting the evaluation process, including by provision of marked vehicles, may have influenced the responses of interviewees, many of whom were indirect beneficiaries of UNICEF support. Furthermore, the in-field evaluation was conducted by rapid appraisal with the researchers spending a very short time in the country. Thus the impressions we gained must be regarded as a snapshot, raising questions for further exploration by researchers based in Ghana, who are familiar with the languages and culture throughout the country and who can spend more time studying health systems issues with the depth required.

## Conclusions

Ghana has made efforts to build a community-based delivery platform for the introduction of iCCM, an effective intervention for child survival. However, important contextual and health system factors, notably poor retention of key health workers, absence of incentives for CBAs who operate outside of the formal health system, and inadequate funds to replenish equipment and transport, threaten its sustainability. Policymakers and key donors need to explore innovative and sustainable mechanisms [53] to secure this programme as part of a government owned and government led strategy.
